# Blood‐based assessment of oxidative stress, inflammation, endocrine and metabolic adaptations in eventing horses accounting for plasma volume shift after exercise

**DOI:** 10.1002/vms3.1409

**Published:** 2024-03-22

**Authors:** Johanna Giers, Alexander Bartel, Katharina Kirsch, Simon Franz Müller, Stephanie Horstmann, Heidrun Gehlen

**Affiliations:** ^1^ Equine Clinic Internal Medicine Freie Universität Berlin Berlin Germany; ^2^ Institute for Veterinary Epidemiology and Biostatistics Freie Universität Berlin Berlin Germany; ^3^ Department Sensors and Modeling Leibniz Institute for Agricultural Engineering and Bioeconomy (ATB) Potsdam Germany; ^4^ Laboklin Veterinary Laboratory Diagnostics Bad Kissingen Germany; ^5^ German Olympic Committee for Equestrian Sports (DOKR) Warendorf Germany

**Keywords:** cross‐country, eventing, oxidative stress, performance diagnostics, serum amyloid, sport horses, superoxide‐dismutase

## Abstract

**Background:**

After submaximal exercise, blood values of eventing horses show physiological reactions.

**Objectives:**

This prospective longitudinal study investigated blood parameters in 20 elite eventing horses before and after two–four‐star cross‐country rides.

**Methods:**

Using a mixed model adjusting for plasma volume shift, we assessed exercise‐dependent parameters and compared blood values with reference ranges for healthy horses at rest.

**Results:**

Following exercise, cortisol, triiodothyronine (T3) and thyroxine (T4) showed short‐term increases, and superoxide‐dismutase showed a small short‐term increase. Hepatic values showed short‐term (haemoglobin [HGB], globulins) or sustained increases (bilirubin, glutamate dehydrogenase, alanine aminotransferase). Digestion‐related parameters showed small short‐term increases (α‐amylase, triglycerides) or decreases (cholesterol, DGGR‐lipase), apparent through plasma shift adjustment. Zinc decreased in the short term, and iron showed a delayed decrease. White blood cell count increased persistently after training, whereas serum amyloid A remained unchanged.

**Conclusions:**

Exercised eventing horses had consistently elevated HGB and cortisol levels 10 and 30 min after submaximal exercise, exceeding the reference ranges for healthy horses at rest. Exercise activates the hypothalamic–pituitary–adrenocortical and hypothalamic–pituitary–thyroid axes. Antioxidant activity was observed. Increased energy requirements led to the mobilization of energy reserves, and a sustained increase in liver enzymes indicated hepatocellular injury. Mild haemolysis suggested increased muscle metabolism, whereas signs of inflammation were subtle. Further research is needed to identify which horses deviate from mean values.

## Graphical abstract

Graphical Abstract: After submaximal exercise (two–four‐star cross‐country rides), trained eventing horses showed consistently elevated HGB and cortisol levels, above the reference ranges for healthy horses at rest. However, physiological changes also occur within the reference ranges. Hormonal axes are activated, and there is antioxidant activity. A sustained increase in liver enzymes suggests hepatocellular injury. Signs of haemolysis reflect increased muscle metabolism.

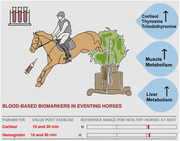

## 1 INTRODUCTION

Fatal injuries in eventing competitions have raised the question of whether the sport is fundamentally dangerous to the health of competing horses. A recent assessment of the physiological responses of blood‐based markers to cross‐country exercise provides objective information on the health, stress levels and performance of competing horses.

Ensuring energy supply during exercise involves endocrine responses. Cortisol modulates physical and mental performance demands on the hypothalamic–pituitary–adrenocortical (HPA) axis (Fazio et al., 2015; Ferlazzo et al., 2018, 2020; González et al., 1998; Kowalik & Tomaszewska, 2018; Liburt et al., 2013). Ferlazzo et al. (2018) resumed that in different equine disciplines, the role of the hypothalamic–pituitary–thyroid (HPT) axis and a connection to the HPA axis in response to physical exercise are suspected but not yet understood. Iodothyronines, especially T3, significantly impact exercise metabolism, accelerating the conversion of carbohydrates, proteins and fats at the cellular level (Ferlazzo et al., 2018, 2020). Although T4 can only be formed in the thyroid gland, T3 can also be synthesized extrathyroidally in target tissues such as muscles (Fazio et al., 2015). T3 increases after standardized exercise tests in standardbred racehorses (Fazio et al., 2022) and maximal speed tests in thoroughbred racehorses (González et al., 1998). A T4 increase 30 min after exercise has only been observed in isolated cases after different exercise intensities, whereas T4 levels 24 h after exercise are often altered (Ferlazzo et al., 2020).

Oxidative stress, which arises from an imbalance between the production and neutralization of reactive oxygen species (ROS) (Siqueira et al., 2014; Williams, 2016), can lead to muscle damage, fatigue and decreased performance (Siqueira et al., 2014; Williams et al., 2004). To reduce the harmful effects of ROS, antioxidant defence systems play a crucial role, consisting of enzymatic components like superoxide‐dismutase (SOD) and non‐enzymatic components like tocopherols (Siqueira et al., 2014). Williams and Burk (2012) found that markers of antioxidant status were upregulated in eventing horses after difficult exercise, which they attribute to increased scavenging of ROS.

Metabolic stress is caused by the increased energy requirements of physical exercise. Cross‐country exercise places aerobic and, as difficulty increases, increasingly anaerobic metabolic demands on the equine body (Amory et al., 1993; Muñoz et al., 1999; Williams & Burk, 2012). Anaerobic energy can be supplied through anaerobic glycolysis from glucose and through the creatine–phosphate–ATP pathway (McGowan et al., 2002). Conversely, aerobic energy supply relies on aerobic glycolysis from carbohydrates or beta‐oxidation from fats (McGowan et al., 2002). Metabolic stress can also lead to an accumulation of metabolic products, which may contribute to fatigue and muscle exhaustion (Hureau et al., 2022; McGowan et al., 2002; Mihelic et al., 2022).

Inflammation is another possible stress factor for horses in eventing competitions (Arfuso et al., 2020; Munsters et al., 2014). Although inflammatory responses are a natural part of adaptive mechanisms (Kellmann et al., 2018), overtraining can lead to excessive inflammatory responses that negatively impact the athlete (Witkowska‐Pilaszewicz, Bąska, 2019; Witkowska‐Pilaszewicz, Żmigrodzka^2019^). Serum amyloid A (SAA), a highly sensitive but non‐specific marker of inflammation, has been extensively studied in horses for detection of different subclinical pathologies, as well as for monitoring the response of treatment (Kowalik & Tomaszewska, 2018; Long & Nolen‐Walston, 2020; Turlo et al., 2016; Valle et al., 2015; Witkowska‐Pilaszewicz, et al., 2019). Valle et al. (2015) did not observe an increase in SAA but did observe an increase in leukocyte levels after a national Italian eventing competition in eight warmblood horses, although SAA appears to be more sensitive to inflammation than white blood cell (WBC) counts (Long & Nolen‐Walston, 2020).

The present study provides benchmarks for trained and healthy horses and various blood parameters after submaximal exercise in modern high‐level eventing competitions. Our approach accounts for plasma volume shifts during exercise, enhancing the interpretation of blood values. A better understanding of different physiological pathways and comparison with valid reference ranges for healthy horses at rest will contribute to improved monitoring of the performance, well‐being and health of eventing horses.

## 2 MATERIALS AND METHODS

Riders and owners were informed in writing about the study conditions and gave their written consent for voluntary and free participation. The study was not classified as an animal experiment but was registered with the regulatory state office of Berlin (1‐02.04.40.2022.VG006). Blood samples were collected by veterinarians involved in routine performance diagnostics as part of the ‘performance monitoring programme’ of the German Olympic Committee for Equestrian Sports (DOKR). All horses were clinically and echocardiographically examined by DOKR project veterinarians and declared healthy before participation. None of the horses were treated with treatment techniques or medication that contravened the Fédération Equestre Internationale (FEI) (2022a, 2022b, 2022c, 2022d) rules for international eventing competitions. The riders also confirmed that the horses did not receive any intravenous infusions during the competitions. Only authorized oral electrolyte substitution was administered in individual cases.

### 2.1 Study design

Materials and methods in this study are consistent with those used in Giers et al. (2023), as the parameters were measured in the same blood samples.

The study took place between March and September 2022 at 14 international two‐ to four‐star level eventing competitions at five different venues in Germany and Poland. Nine riders with 20 horses participated in the study.

All horses were sampled at 2–5 competitions throughout the season, resulting in 4–20 samples per horse. Before and after each test, the horses were subjected to mandatory veterinary checks by official FEI veterinarians and were declared ‘fit to compete’.

#### 2.1.1 Horses

The subjects of the present longitudinal observational study were horse–rider combinations that were monitored as part of the ‘performance monitoring programme’ project of the DOKR.

Horses in the study were aged between 7 and 15 years, with an average age of 11. The population consisted of 10 mares and 10 geldings. The horses belonged to nine different warmblood breeds (Table 1). Relevant horse identification information was obtained from the FEI database 2022b.

**TABLE 1:** Characteristics of horses participating in the study.

Horse ID	Age (years)	Breed	Sex	International experience^†^	Competitions during season 2022^‡^
1	7	Holsteiner	Mare	2	4
2	7	Oldenburger	Mare	5	6
3	7	Hanoverian	Mare	3	6
4	8	Oldenburger	Mare	11	7
5	8	Westphalian	Gelding	4	4
6	9	Hanoverian	Gelding	11	6
7	9	German Sport Horse	Mare	11	4
8	10	Polish Horse Breeders Association	Mare	17	7
9	10	Irish Sport Horse	Gelding	18	4
10	11	Hanoverian	Gelding	15	5
11	11	Holsteiner	Gelding	20	6
12	12	Stud Book du Cheval Selle Français	Mare	22	6
13	12	Hanoverian	Gelding	31	6
14	12	Irish Sport Horse	Gelding	28	6
15	12	Hanoverian	Mare	17	7
16	14	Hanoverian	Gelding	22	6
17	14	Holsteiner	Gelding	35	3
18	15	Hanoverian	Mare	56	5
19	15	Rheinlander	Mare	24	2
20	15	Hanoverian	Gelding	40	8

^†^ Number of starts in international eventing competitions before start of season 2022.

^‡^ Number of international eventing competitions during season 2022.

#### 2.1.2 Riders

The riders were aged between 21 and 39. The average age was 28. There were three male and five female riders in the study. In order to compete at their respective test level, all of the riders achieved the appropriate performance level through placings in lower classes to ensure they had a comparable level of performance.

#### 2.1.3 Training schedules

The horses had been individually prepared for the competition by their riders and trainers. Each training schedule includes dressage, jumping and cross‐country training, as well as basic endurance training, light work days and paddock or pasture‐only days. The specific training schedule for each horse was neither reported nor standardized. Neither feeding nor water intake were recorded or standardized during the season, which had an effect on the interpretability of the digestion‐associated parameters.

#### 2.1.4 Exercise

An eventing competition consists of three subtests: dressage, jumping and cross‐country. This study focuses on the cross‐country test, which was either the second or third test in the eventing competition, depending on the test format.

Nine rides at a 2‐star level, 31 rides at a 3‐star level and 15 rides at a 4‐star level were sampled in accordance with the rules of the FEI for international eventing competitions (2022a). The specific test requirements for the cross‐country tests are set out in Table 2. An average of 9.2 penalty points was achieved in the cross‐country test. Horse falls led to the disqualification in two rides, and in one ride, a rider chose to retire. Information on diet, water intake, and additional exercise during the competition days was not recorded.

**TABLE 2:** Cross‐country competition information, as well as distance, speed, environmental parameters, ground and altitude profile.

Competition	Level	Week	Venue	Horses *N* =	Distance [m]	Speed required [m/min]	Temperature [°C]	Ground	Altitude profile	Weather
1	CCI3*‐S	12	A	11	3401	550	13	Normal	Flat	Cloudy
2	CCI3*‐S	15	B	7	3007	520	16	Normal	Intermediate	Sunny
3	CCI2*‐S	16	A	2	3000	520	16	Normal	Flat	Cloudy
4	CIC2*‐L	18	C	1	3787	520	16	Deep	Hilly	Sunny
5	CCI2*‐S	18	C	2	3085	520	18	Normal	Hilly	Cloudy
6	CCI4*‐S	18	C	7	3705	570	20	Deep	Hilly	Sunny
7	CCI4*‐S	24	A	3	3772	570	29	Normal	Flat	Sunny
8	CIC3*‐L	25	A	3	4455	550	26	Normal	Flat	Sunny
9	CCI3*‐S	25	A	3	3364	550	25	Normal	Flat	Sunny
10	CCI2*‐S	30	D	2	2661	520	20	Normal	Hilly	Cloudy
11	CCI3*‐S	30	D	6	3538	550	20	Normal	Hilly	Cloudy
12	CCI2*‐S	35	E	1	3087	520	28	Normal	Flat	Sunny
13	CCI3*‐S	35	E	2	3470	550	28	Normal	Flat	Sunny
14	CCI4*‐S	35	E	5	3580	570	28	Normal	Flat	Sunny

Abbreviations: ‘‐L’, long for‐mat; ‘‐S’, short format; CCI, Concours Complet International; CIC, Concours International Combiné.

#### 2.1.5 Sampling times

The collection of samples was carried out at different time points, including the morning before exercise (Pre), 10 min (10 min) and 30 min (30 min) after the completion of the exercise and 24 h after the Pre sample (next morning). The pre‐exercise and next morning samples were collected between 4:00 and 7:30 AM, whereas the 10 and 30 min samples were collected between 9:00 AM and 5:00 PM, depending on the different starting times of the horses. Consequently, the time intervals between the pre‐ and post‐exercise samples, as well as between the 30 min samples and the next morning samples, varied according to the horses’ start times. The time between 30 min after exercise and the next morning ranged from 11 to 21 h, whereas the time between the pre‐sample and the next morning was consistently 24 h (±1 h).

### 2.2 Sample collection

In the morning before and in the morning after the cross‐country test, the blood samples were taken in the horses’ stables so that all blood samples could be taken within 30 min. For the samples taken 10 and 30 min after the finish, the time at which the respective horse crossed the finish line was observed, and the respective test time was determined. The blood samples were then obtained either in the finish area or in the stable area, depending on where the horses were found. Blood samples were drawn from the horses’ jugular veins, and the puncture site was disinfected with 1‐propanol. Venous blood was collected using a Vacutainer system equipped with 20G needles and polyethylene terephthalate tubes. During each blood collection, first a serum tube and then an EDTA tube were filled. After each blood collection, EDTA whole blood tubes were stored in a refrigerator at +5°C within 5 min, whereas serum gel tubes were left at room temperature for 30–60 min until clotting occurred. A portable centrifuge, model EBA 200 from Andreas Hettich GmbH & Co. KG, was used to centrifuge the serum tubes at 1000 × *g* for 10 min before transferring the serum to uncoated plastic tubes, which were immediately frozen at −20°C. The blood samples were processed in a mobile laboratory in the finish area of the cross‐country course or in the stable area.

#### 2.2.1 Sample storage and transport to testing laboratory

The blood samples were taken, stored and processed as described in Giers et al. (2023). The samples were transported in electric freezers that maintained a constant temperature of +5 and −20°C, respectively. All samples were transported from the venue to the in‐house laboratory of the German Equestrian Federation in Warendorf within 48 h, where the refrigerated EDTA whole blood samples were measured immediately. The serum samples were stored at 20°C for a maximum of 8 weeks until they were sent frozen to the external testing laboratory, LABOKLIN GmbH & Co.KG.

#### 2.2.2 Blood parameters

In addition to parameters discussed in our previous work (Giers et al., 2023), this work focuses on parameters measured in the areas of inflammation, oxidative stress, hormones, liver, digestion and trace elements (Table 3). Leucocytes (WBC), superoxide dismutase (SOD), cortisol and haemoglobin (HGB) were determined at all time points, whereas parameters α‐amylase, alkaline phosphatase (AP), γ‐glutamyl transferase (GGT), glutamate dehydrogenase (GLDH), DGGR‐lipase, bilirubin, cholesterol (CHOL), triglycerides (TRIG), globulines (GLOB), SAA, iron, copper, zinc, selenium, vitamin E (Vit. E), T3 and T4 were not determined 10 min post‐exercise but at all other measurement time points.

**TABLE 3:** Blood‐based biomarker and associated categories.

Category	Biomarker
Hormones	T3, T4, cortisol
Antioxidative markers	SOD, vitamin E
Hepatic values	HGB, bilirubin, GGT, GLDH, globulines, ALT
Digestion	α‐Amylase, AP, cholesterol, triglycerides, DGGR‐lipase
Trace elements	Iron, copper, selenium, zinc
Inflammatory markers	WBC, SAA

Abbreviations: AP, alkaline phosphatase; GGT, γ‐glutamyl transferase; GLDH, glutamate dehydrogenase; HGB, haemoglobin; SAA, serum amyloid A; SOD, superoxide‐dismutase; WBC, white blood cell.

#### 2.2.3 Measurement techniques

WBC and HGB were determined using laser flow cytometry and laminar flow impedance in a ProCyte DX haematology analyser from IDEXX Laboratories Inc. Serum samples were further processed in the investigation laboratory (LABOKLIN). T3 and T4 were measured with a chemiluminescence assay (LIA), T3 by ADVIA Centaur XPT 2000 (Siemens), and T4 by Immulite 2000 (Siemens). Vit. E analysis was performed by high‐performance liquid chromatography (HPLC) in an HPLC LC‐20 (Shimadzu). An atomic absorption spectrometry (AAS) Zenit 650 P (Analytik Jena GmbH), which measures with AAS, was used for the selenium analyses. The concentration of SOD was determined using a manual enzymatic reaction with materials from the manufacturer, Cayman Chemical. All other blood chemical parameters were measured photometrically or potentiometrically in a cobas 8000 (Roche).

### 2.3 Missing values

In 54 of 55 rides, sample sets were complete. The ‘next morning’ sample is missing in one set because the rider refused to take the sample in a stressful situation. In two blood collections, the serum tube was lost after collection. In one blood collection, the amount of blood was insufficient to fill all sample tubes, resulting in the absence of certain values for this measurement.

### 2.4 Data analysis

Statistical analyses were performed using Jamovi (Version 2.3) (‘The jamovi project (2022) jamovi (Version 2.3) [Computer Software].’). Boxplot, histogram and *Q*–*Q* plot were used to visually check the normality of the data. Due to non‐normality, GGT, GLDH, SOD, T3, TRIG and α‐amylase values were log‐transformed. SAA values were log1p‐transformed.

#### 2.4.1 Plasma volume shift adjustment

Blood values are expressed as concentrations, which represent an amount per volume. During exercise, intravascular blood volume changes due to splenic contraction, plasma volume loss and dehydration. Furthermore, the actual amount of blood parameters (e.g. cells, enzymes and metabolites) changes. Separating the volume‐related changes from the quantity‐related changes can contribute to a more accurate understanding of the effects of exercise on specific parameters and specific body systems.

To separate volume effects from quantity effects, we used the adjustment proposed in Giers et al. (2023) for the plasma volume shift during exercise. The albumin concentration is used as a marker for the concentration effect that occurs due to the plasma volume shift from intra‐ to extravascular (Assunção et al., 2019; EclinPath, 2023; Giers et al., 2023).

The observed values 10 and 30 min after the end of exercise were adjusted by a calculated factor, whereas the values pre‐exercise and the next morning remained unadjusted. The following formula was used for the adjustment:

(1) $$\begin{array}{ccc} & & \mathrm{adjusted}\;\mathrm{value}=\mathrm{measured}\;\mathrm{valu}e_{\mathrm{time}\;\mathrm{point}\;x}/\\ & & \;\left(1+\mathrm{plasma}\;\mathrm{volume}\;\mathrm{loss}\;{\left(\%\right)}_{\mathrm{time}\;\mathrm{point}\;x}\right) \end{array}$$

#### 2.4.2 Mixed model

Estimated marginal means (EMMs) were calculated using a mixed model. Time point was used as a fixed effect, and ‘horse’ nested in ‘rider’ as well as ‘competition’ nested in ‘event’ were used as random effects to account for clustering. The normality of the residual distribution was assessed through a normal likelihood plot of the residuals.

The mixed model was employed for both the observed and adjusted datasets. Figures display EMMs (with confidence intervals) for the adjusted values, represented by light grey dots with dark grey bars. In this study, 95% confidence intervals were computed, and the significance threshold was established at 5%. The *p*‐values presented in the figures indicate the difference in EMM between the ‘Pre’ time point and the corresponding time point, and these values were computed using the plasma shift–adjusted data.

For SAA, no adjusted EMMs are shown in Figure 6b, as they do not differ from the EMM without adjustment.

We investigated whether the parameters of interest showed exercise‐related increases or decreases, quantified the magnitude of these changes and assessed how much their EMMs deviated from the reference ranges for healthy horses at rest. We calculated the relative changes in the unadjusted EMMs (EMM_unadjusted_) at the different time points.

Blood values outside the established reference ranges are usually highlighted in laboratory reports to facilitate prompt identification by veterinarians. Therefore, we counted the instances where values fell outside the reference ranges to assess the frequency of such deviations and their potential clinical significance.

## 3 RESULTS

The observed descriptive data (Table S1) as well as the EMMs of the unadjusted values (Table S2) and their 95% confidence intervals for the respective parameters are presented in the supplements.

### 3.1 Hormones

**FIGURE 1:**
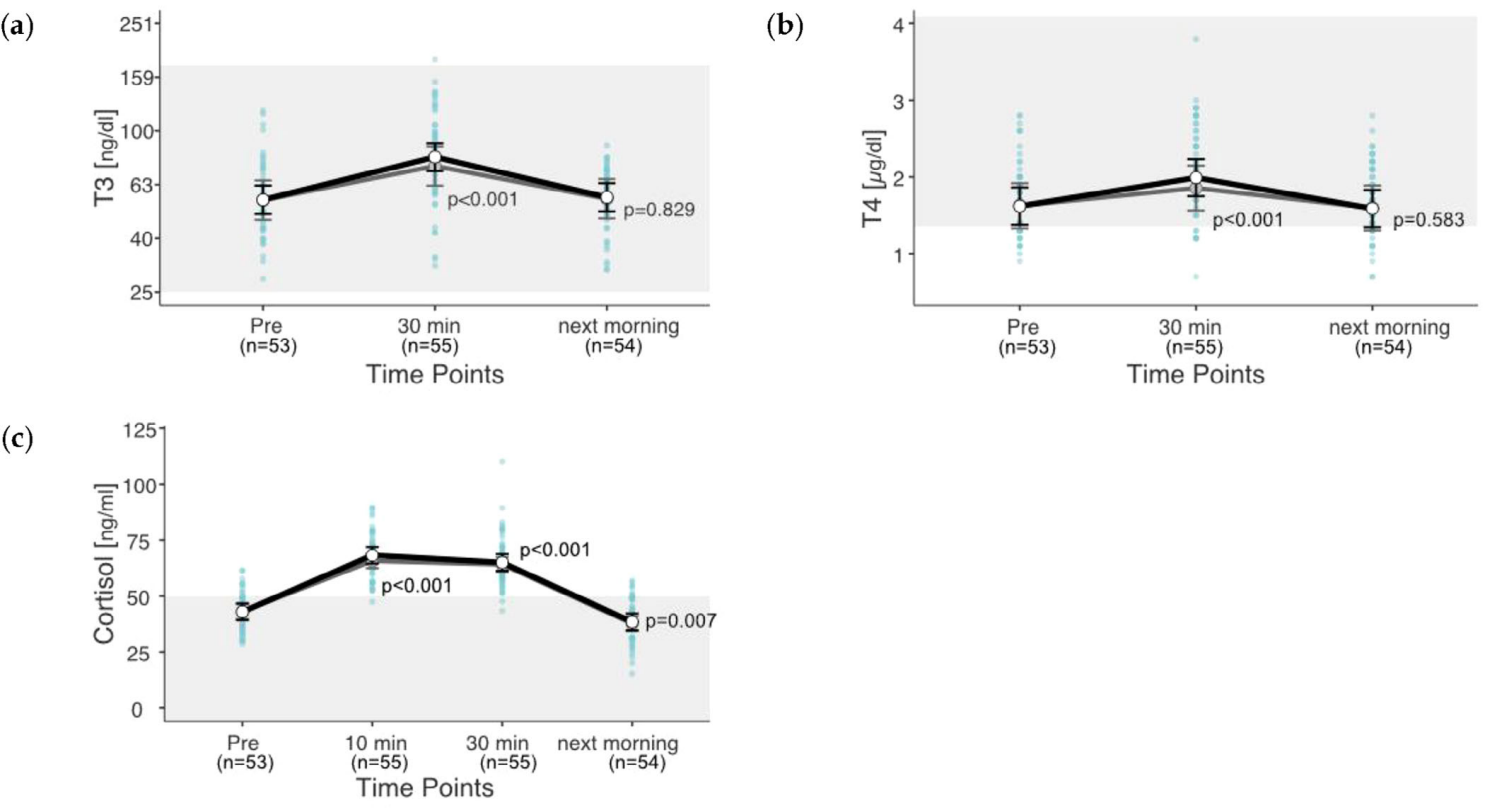
(a–c) Changes **in hormones after exercise**. Estimated marginal means (EMMs) (white dots) and their 95% confidence intervals (black whiskers) of blood parameters at measured time points. Grey dots and whiskers represent EMM and 95% confidence intervals when 10 and 30 min post‐exercise values were adjusted for plasma shift. *p*‐Values apply to the difference between Pre and the respective time point. Blue dots represent observed values. The grey box symbolizes the current reference range for healthy horses at rest of the related blood parameters. Mixed model of blood values against time points. Two random effects (‘horse’ in ‘rider’; ‘competition’ in ‘event’) to adjust for the hierarchical structure of the data.

For the thyroid hormones T3 and T4, ranges between 28.1 and 185.1 ng/dL and 0.7–3.8 µg/dL were measured, with, respectively, 0.6% of the T3 values outside the reference range and 13.0% below the reference range for T4. A maximum cortisol level of 121 ng/mL was measured. Overall, 57.5% of the cortisol values are above the reference range. Cortisol values were above the reference range in 54 of 55 rides. T3 was above the reference range in only one ride, whereas T4 was below the reference range in 11 rides. The hormone concentrations of T3 (*p* = 0.006), T4 (*p* < 0.001) and cortisol (*p* < 0.001) change significantly through exercise. T4 values pre‐exercise and the next morning are within the lower range of the reference (Figure 1b). For cortisol, the EMMs are above the reference range for time points 10 and 30 min post‐exercises. T3 is 44% higher 30 min post‐exercise than pre‐exercise (Figure 1a). T4 is 25% higher 30 min post‐exercise compared to pre‐exercise (Figure 1b). The next morning, only a 2% increase in T3 remained. Cortisol is still 60% elevated 30 min post‐exercise (Figure 1c). In contrast, the next morning, the cortisol level was 12% lower than pre‐exercise.

### 3.2 Antioxidative markers

**FIGURE 2:**
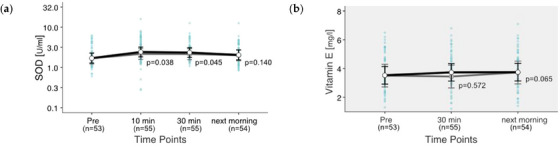
(a and b) Changes in antioxidative markers after exercise. Estimated marginal means (EMMs) (white dots) and their 95% confidence intervals (black whiskers) of blood parameters at measured time points. Grey dots and whiskers represent EMM and 95% confidence intervals when 10 and 30 min post‐exercise values were adjusted for plasma shift. *p*‐Values apply to the difference between Pre and the respective time point. Blue dots represent observed values. The grey box symbolizes the current reference range for healthy horses at rest of the related blood parameters. Mixed model of blood values against time points. Two random effects (‘horse’ in ‘rider’; ‘competition’ in ‘event’) to adjust for the hierarchical structure of the data.

SOD and Vit. E were measured as markers for oxidative stress. SOD ranged from 0.254 to 14.646 U/mL. For Vit. E, the values were between 1.3 and 7.2 U/L. For SOD, the laboratory (LABOKLIN GmbH) does not provide a validated reference range in horses. Exercise has no significant influence on the values of SOD (*p* = 0.137), but on the values of Vit. E (*p* = 0.041). All EMMs are within the reference range for Vit. E. SOD shows significant increases in value before exercise compared to 10 and 30 min post‐exercise. SOD is estimated to be 38% higher 30 min after exercise and 20% higher the next morning compared to Pre, although the deviation is no longer significant the next morning (Figure 2a). Vit. E concentration shows a 6% increase 30 min after the end of exercise and an 8% increase in the morning after exercise (Figure 2b).

### 3.3 Hepatic values

**FIGURE 3:**
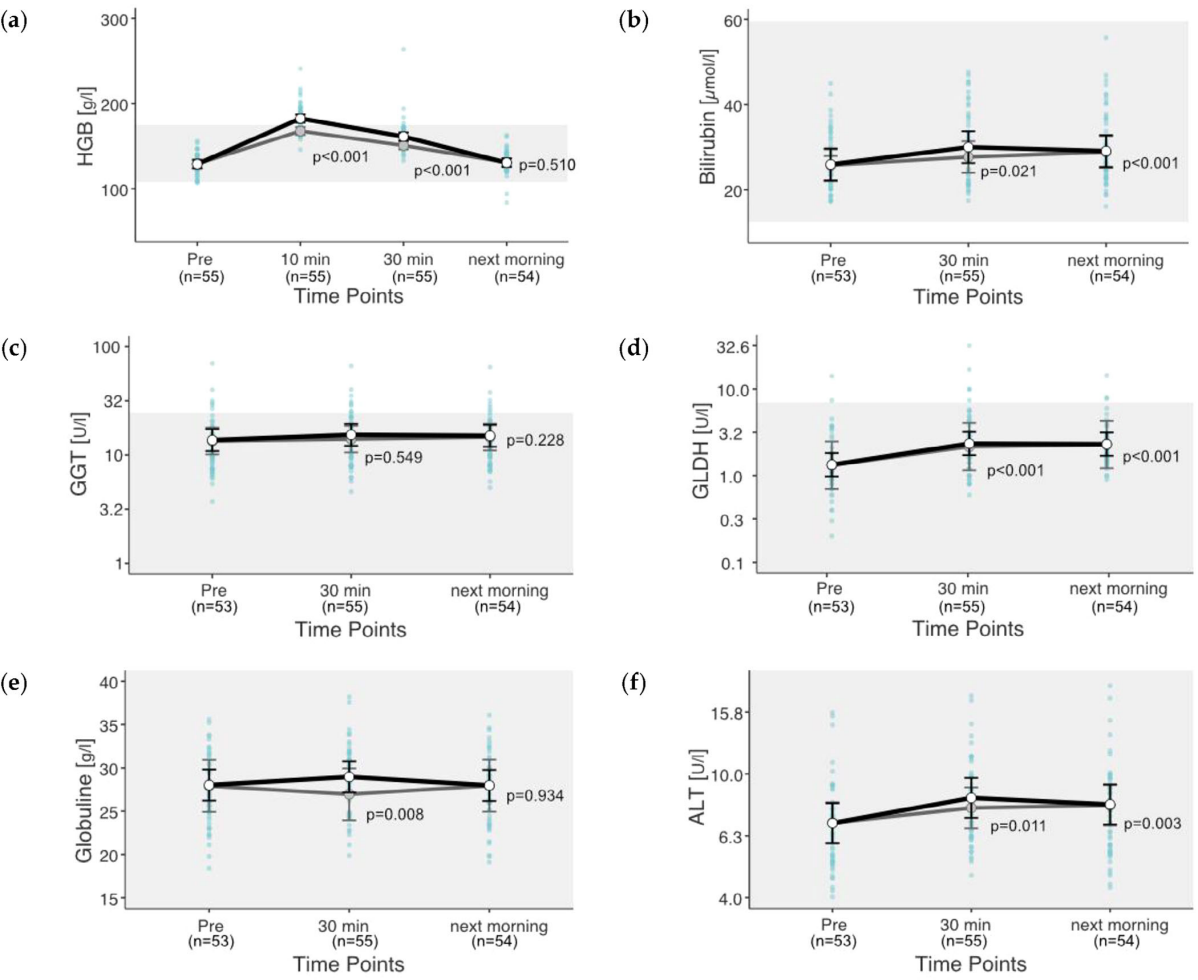
(a–f) Changes **in hepatic values after exercise**. Estimated marginal means (EMMs) (white dots) and their 95% confidence intervals (black whiskers) of blood parameters at measured time points. Grey dots and whiskers represent EMM and 95% confidence intervals when 10 and 30 min post‐exercise values were adjusted for plasma shift. *p*‐Values apply to the difference between Pre and the respective time point. Blue dots represent observed values. The grey box symbolizes the current reference range for healthy horses at rest of the related blood parameters. Mixed model of blood values against time points. Two random effects (‘horse’ in ‘rider’; ‘competition’ in ‘event’) to adjust for the hierarchical structure of the data.

HGB ranged between 84 and 264 g/L, GGT between 3.7 and 69.9 U/L and GLDH between 0.2 and 31.5 U/L. For ALT, bilirubin, and GLOB, all values are within the respective reference range. Overall, 29.2% of the HGB, 12.3% of the GGT and 4.3% of the GLDH values were above the respective reference range. HGB was above the reference in 48 out of 55 rides and below the reference in 2 rides. GGT and GLDH were elevated in 8 and 6 out of 55 rides, respectively. The liver‐related parameters HGB, bilirubin, GLDH, globulins and ALT responded to exercise (*p *< 0.05), but GGT did not (*p* = 0.482). Only HGB 10 min after the end of the exercise is above the reference range. The 25% HGB increase 30 min post‐exercise is fully recovered the next morning (2% increase) (Figure 3a). In the case of bilirubin, 13% of the 15% increase after 30 min still remains the next morning (Figure 3b). Of the 12% increase in GGT concentration 30 min post‐exercise, 9% increase is still present the next morning (Figure 3c). In the case of GLDH, 30 min post‐exercise, a 77% increase was observed, which remained until the next morning (Figure 3d). ALT showed an increase between 22% (30 min) and 16% (next morning) (Figure 3f).

### 3.4 Digestion‐associated parameters

**FIGURE 4:**
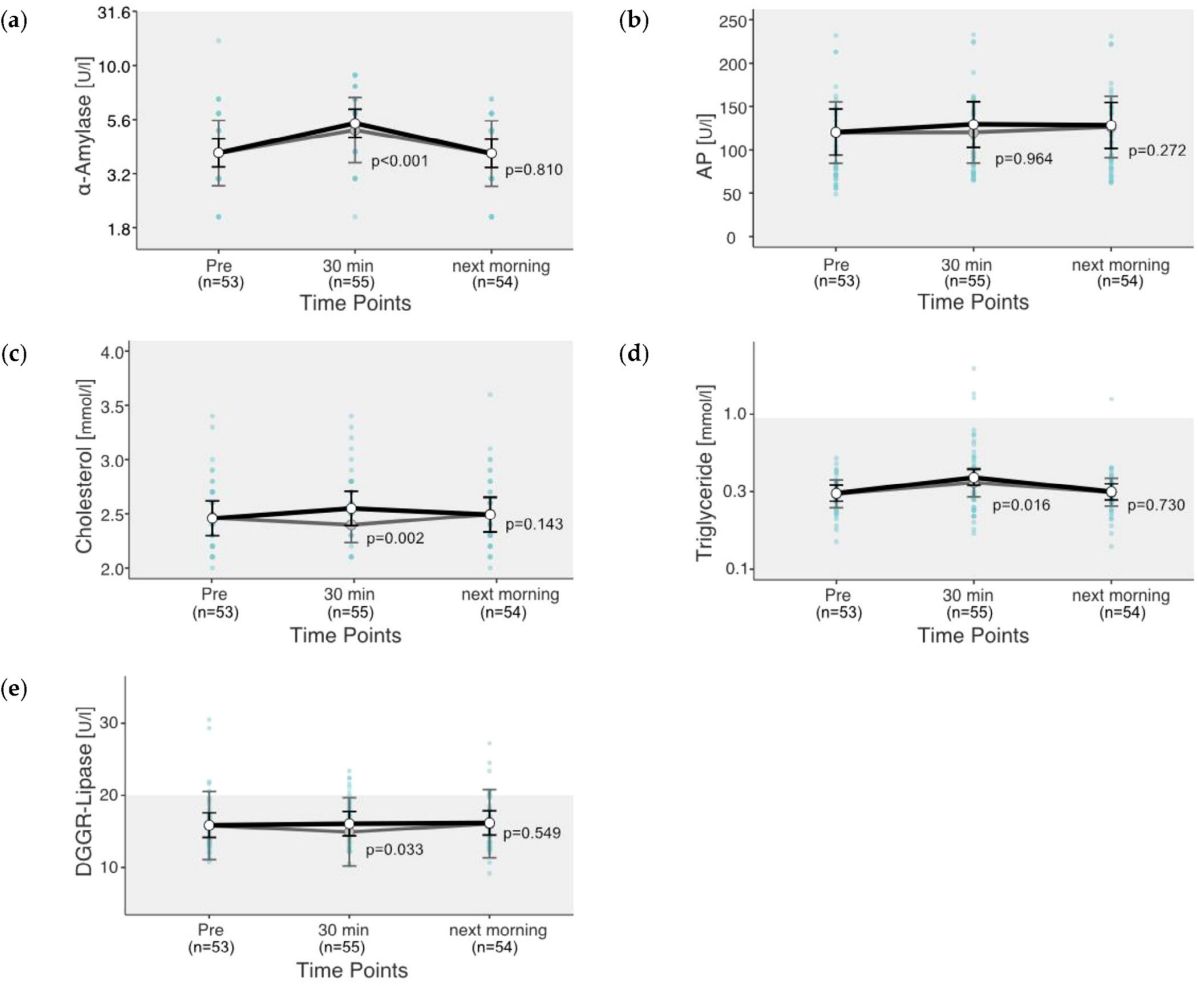
(a–e) Changes in digestion‐associated parameters after exercise. Estimated marginal means (EMMs) (white dots) and their 95% confidence intervals (black whiskers) of blood parameters at measured time points. Grey dots and whiskers represent EMM and 95% confidence intervals when 10 and 30 min post‐exercise values were adjusted for plasma shift. *p*‐Values apply to the difference between Pre and the respective time point. Blue dots represent observed values. The grey box symbolizes the current reference range for healthy horses at rest of the related blood parameters. Mixed model of blood values against time points. Two random effects (‘horse’ in ‘rider’; ‘competition’ in ‘event’) to adjust for the hierarchical structure of the data.

For the digestion‐associated parameters, α‐amylase, AP and CHOL showed only values within the reference ranges. Overall, 2.5% of the TRIG values were above the reference range, with a maximum of 1.97 mmol/L. For DGGR‐Lipase, 11.7% of the values were above the reference range, with a maximum of 30.5 U/L. Although TRIG were elevated in only four rides, lipase was above the reference range in 14 of 55 rides. Exercise changed the values of all digestion‐associated parameters (*p *< 0.05), except AP (*p* = 0.456). The EMMs of the digestion‐associated parameters were all within the respective reference ranges (Figure 4a,e). The strongest percentage changes 30 min post‐exercise showed TRIG with a 26% increase (Figure 4d). α‐Amylase increased by 25% at 30 min post‐exercise (Figure 4a). The next morning, AP showed the largest increase (7%) among the digestion‐associated parameters.

### 3.5 Trace elements

**FIGURE 5:**
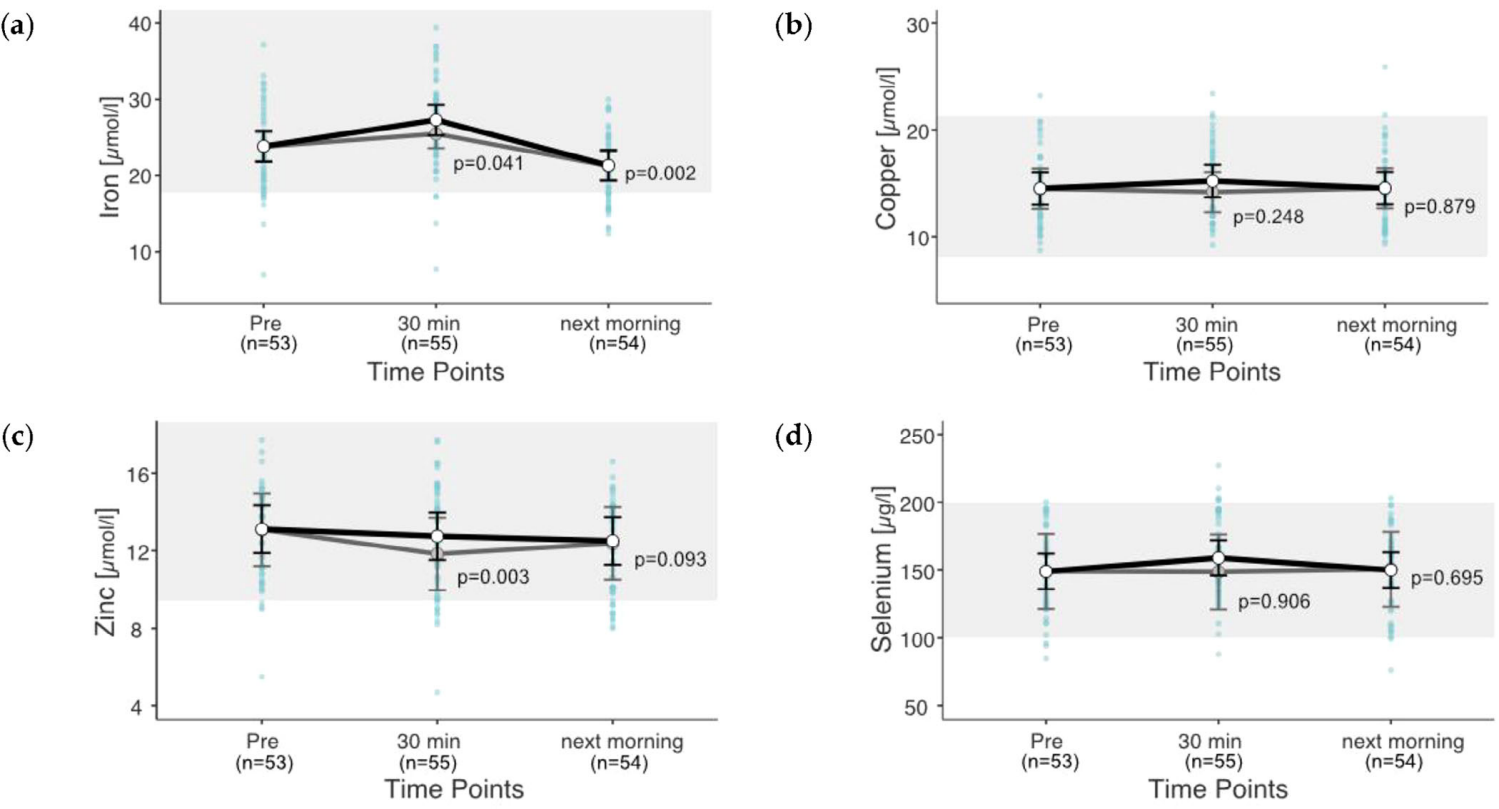
(a–d) Changes in trace elements after exercise. Estimated marginal means (EMMs) (white dots) and their 95% confidence intervals (black whiskers) of blood parameters at measured time points. Grey dots and whiskers represent EMM and 95% confidence intervals when 10 and 30 min post‐exercise values were adjusted for plasma shift. *p*‐Values apply to the difference between Pre and the respective time point. Blue dots represent observed values. The grey box symbolizes the current reference range for healthy horses at rest of the related blood parameters. Mixed model of blood values against time points. Two random effects (‘horse’ in ‘rider’; ‘competition’ in ‘event’) to adjust for the hierarchical structure of the data.

Overall, 16.0% of the iron values were below the reference with a minimum of 7 µmol/L. Overall, 3.7% of the copper values were above the reference range, with a maximum of 25.9 µmol/L. Overall, 10.5% of the zinc values were below the reference, at a minimum of 4.7 µmol/L. For selenium, 3.7% of the values were below and 4.3% above the reference range, with a range of 76.3–227 µg/L. Zinc was below the reference in 14 out of 55 rides, and iron in 19 of 55 rides. Exercise significantly changed the concentrations of iron (*p* < 0.001) and zinc (*p* = 0.013). Exercise did not significantly change the concentrations of copper (*p* = 0.353) and selenium (*p* = 0.865). All estimated trace element values are within the respective reference ranges (Figure 5a,d). The largest percentage changes 30 min post‐exercise showed iron (14% increase) and selenium (7% increase). The next morning, iron showed the strongest changes, with an 11% decrease compared to pre‐exercise.

### 3.6 Inflammatory markers

**FIGURE 6:**
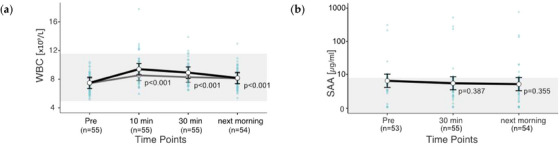
(a and b) **Changes in inflammatory markers after exercise**. Estimated marginal means (EMMs) (white dots) and their 95% confidence intervals (black whiskers) of blood parameters at measured time points. Grey dots and whiskers represent EMM and 95% confidence intervals when 10 and 30 min post‐exercise values were adjusted for plasma shift. *p*‐Values apply to the difference between Pre and the respective time point. Blue dots represent observed values. The grey box symbolizes the current reference range for healthy horses at rest of the related blood parameters. Mixed model of blood values against time points. Two random effects (‘horse’ in ‘rider’; ‘competition’ in ‘event’) to adjust for the hierarchical structure of the data.

Leukocytes (WBC) and SAA were measured as inflammatory markers. WBC ranged from 5.18 to 17.8 × 109/L. SAA values lay between 0.1 and 758.6 µg/mL. Overall, 5.5% of the WBC values and 20.4% of the SAA values exceeded the reference range. Although WBC was elevated in 6 out of 55 rides, SAA had values above the reference in 21 rides, of which 11 were measured at rest. WBC responded to the exercise (*p* < 0.001), whereas SAA did not (*p* = 0.586). WBCs are well within the reference range at all time points (Figure 6a). For SAA, the whiskers are close to or above the upper limit of the reference range at all measurement times (Figure 6b). WBCs react quickly; 30 min after the end of exercise, a 19% increase is present, of which a 9% increase remains the next morning.

In 55 rides, a pre‐ride SAA value of over 200 µg/mL was measured in only two cases. All other pre‐ride SAA values were below 25 µg/mL. The two horses with clearly elevated pre‐ride SAA values both suffered falls during the cross‐country course. Of the 55 rides sampled, these 2 falls were the only horse falls. Both horses were declared ‘fit to compete’ before the ride. (Overall, 100% of the fallen horses had an elevated SAA value of over 200 µg/mL before the ride.)

## 4 DISCUSSION

When a vet looks at a horse's blood values after exercise, the question is whether blood values outside the reference range are pathological or not. In this study, we present data from a large number of exercising horses to show which blood values are regularly outside the reference ranges for healthy horses at rest. In addition, this study shows how inflammation, oxidative stress, metabolic stress, and endocrine responses manifest in most established blood parameters of top‐level eventing horses following modern international cross‐country rides.

Mean values for all parameters measured, except HGB and cortisol, remain within the reference ranges for healthy horses at rest at all time points. Cortisol, T3 and T4 show short‐term increases after exercise, with only cortisol decreasing the following morning. The antioxidant enzyme SOD shows a small short‐term increase. All hepatic values react with short‐term (HGB, globulins) or sustained increases (Bilirubin, GLDH, ALT). Parameters related to digestion react with small short‐term increases (α‐amylase, TG) or decreases (CHOL, DGGR‐lipase), which become apparent through the plasma shift adjustment. Zinc showed a short‐term decrease, and iron showed a delayed decrease. WBC increased persistently after exercise, whereas SAA did not react.

### 4.1 HPA and HPT axes

The short‐term increases in cortisol, T3 and T4 are consistent with some of the literature (Fazio et al., 2020; Ferlazzo et al., 2014; Ferlazzo et al., 2020; González et al., 1998; Kowalik & Tomaszewska, 2018; Williams & Burk, 2012). However, there are also studies on jumping horses where T3 and/or T4 did not respond to exercise (Ferlazzo et al., 2014; Fazio et al., 2015), which could be due to lower exercise duration and intensity. The increase in cortisol indicates activation of the HPA axis (Ferlazzo et al., 2020), whereas the increase in T3 does not necessarily imply activation of the HPT axis, as T3 can also be synthesized extrathyroidally (Fazio et al., 2015). However, the increase in T4 argues for direct thyroidal regulation (Fazio et al., 2015; Ferlazzo et al., 2020) and thus for activation of the HPT in eventing horses during intense exercise, which is consistent with the opinion of Ferlazzo et al. (2020). Fazio et al. (2015) speculated that an increase in T4 concentration may be associated with a higher efficiency of the mechanical work performed by the exercising muscle.

Further, the drop in cortisol the next morning was not observed in all studies (Kowalik & Tomaszewska, 2018; Williams & Burk, 2012). But in the study by Liburt et al. (2013), the cortisol concentration after adrenocorticotropic hormone (ACTH) stimulation also dropped after the short‐term sharp increase. ACTH has also been studied in horses following exercise, with the result that ACTH increases depending on the intensity of the exercise (Ferlazzo et al., 2020). Thus, the cortisol curve measured in our study may indicate ACTH stimulation. To our opinion, cross‐country exercise results in extensive endocrine adaptations, including activation of the HPA and HPT axes.

### 4.2 Oxidative stress and antioxidative defence

The small increase in SOD levels shortly after the end of exercise indicates that ROS production occurs during exercise, leading to the activation of antioxidative defence systems. However, this does not indicate that there is a discrepancy between ROS production and antioxidant systems, which could constitute pathological oxidative stress (Williams & Burk, 2012). But as ROS are difficult to measure due to their short half‐lives, metabolic products and cofactors must be used for measurement (Bollinger et al., 2023). Possible reasons for ROS production are increased oxygen consumption and mechanical muscle stress (Williams & Burk, 2012).

De Moffarts et al. (2005) also measured antioxidant markers in eventing horses but, contrary to our results, considered SOD and Vit. E not to be exercise‐dependent and therefore only measured pre‐exercise values. Williams and Burk. (2012) measured α‐tocopherol pre‐ and post‐exercises and also found a small increase the morning after exercise. In general, the findings of our study are consistent with other studies on eventing horses (Williams, 2016). Our results further support the view of Bollinger et al. (2023) that elevated SOD could be an indicator of antioxidative capacity already being used (e.g. due to insufficient recovery) and not that there is plenty of antioxidant capacity left. We therefore agree with Bollinger et al. (2023) that a low pre‐ride SOD is beneficial over a high pre‐ride SOD, whereby pre‐exercise nutritional supplementation could be influential.

### 4.3 Haemolysis

The observed increases in HGB and bilirubin could indicate haemolysis (EclinPath). The HGB increase could also be due to hemoconcentration (EclinPath), but this would have been corrected through plasma volume loss adjustment. Bilirubin, being a by‐product of HGB degradation, also serves as an indicator of erythrocyte destruction (EclinPath). The increase in iron concentration 30 min after exercise was also observed by Mihelic et al. (2022) after endurance races and would also speak for haemolysis. However, when considering the plasma shift–adjusted values, it becomes clear that the alleged increase is in fact much smaller and has no relevance. Haemolysis after exercise can be caused by mechanical damage to the erythrocytes or a low oxygen content during exercise and is also suspected in endurance horses (Mihelic et al., 2022). Mechanically induced or cytokine‐induced cell damage in the form of an inflammatory reaction in the musculature (Nieman et al., 2005; Smith, 2000) could be the cause of the observed signs of haemolysis. Possibly both – stress on the musculature and mechanical damage to erythrocytes – may contribute a tiny part to the observed effect.

### 4.4 Hepatocellular injury

The increases in the enzymes GGT and GLDH, which persisted until the next morning, could be due to liver cell injury. GLDH increases primarily reflect hepatocellular injury and cholestasis (Satué et al., 2022). GGT, in contrast, is not as liver‐specific as GLDH, and the increase in GGT could therefore also arise from muscle cell injury (Satué et al., 2022). If GGT is released from liver cells, this indicates biliary necrosis or hyperplasia (Satué et al., 2022). The enzyme ALT (synonym GPT) is also elevated one night after exercise, which could also be due to liver or muscle cell damage (EclinPath). The increases in GGT and ALT could be interpreted as signs of muscle microdamage, but the GLDH increase remains implicative of prolonged hepatocellular injury. A possible cause of hepatocellular injury may be limited oxygen supply during exercise (Elkady et al., 2021), as hepatic blood flow is significantly lower directly after intense exercise in horses (Dyke et al., 1998). If the blood flow returns to normal after exercise, a hepatocellular injury may occur (Elkady et al., 2021).

A further explanation might be the overall significantly increased metabolism in the liver, which is required for the provision of energy as well as for the processing of waste substances.

The increase of GGT, GLDH and ALT within the reference ranges is considered to be mild, compared with a fivefold increase above the reference range in mild liver disease (Satué et al., 2022). Other studies have also shown that there is an exercise‐induced increase in liver enzymes without liver disease (McGowan 2008). In endurance horses, the increase in GGT is not as big, but the increase in AST is even bigger than in eventing horses (Mihelic et al., 2022).

### 4.5 Digestion

The enzymes α‐amylase and TG show short‐term increases after exercise. The increased TG might be synthesized by the liver and transported to cells with increased energy demand (EclinPath). α‐Amylase is an enzyme that hydrolyses complex carbohydrates into maltose and glucose (EclinPath) and is secreted by the exocrine pancreas into the small intestine. In the digestive tract, α‐amylase thus indirectly ensures that more glucose can be absorbed into the blood. On the one hand, during cross‐country exercise, α‐amylase might be excreted to digest available complex carbohydrates and provide energy, which could still be measurable after the end of exercise.

As the digestive function of the intestine is slowed down by the activation of the sympathetic nervous system during exercise, the increased α‐amylase activity could, on the other hand, be a sign of extra‐digestive counter‐regulation against post‐exercise hyperglycaemia. In pigs, α‐amylase has the extra‐digestive function of lowering blood glucose levels independently of insulin (Pierzynowski et al., 2017).

If the reference ranges and the range of the measured values are considered, the mean deviations in the digestion‐associated parameters are small.

### 4.6 Inflammation

There are various studies on SAA in horses pre‐ and post‐exercises (Arfuso et al., 2020; Carvalho Filho et al., 2019; Cywinska et al., 2010; Cywinska et al., 2012; Cywinska et al., 2013; Giori et al., 2011; Kowalik & Tomaszewska, 2018; Kristensen et al., 2014; Long & Nolen‐Walston, 2020; Miglio et al., 2020; Piccione et al., 2016; Rajendren et al., 2019; Siqueira et al., 2016; Turlo et al., 2016; Turlo et al., 2015; Valle et al., 2015; Witkowska‐Pilaszewicz et al., 2019; Witkowska‐Pilaszewicz et al., 2019). In our study, SAA levels did not change post‐exercise. Cywinska et al. (2013) found an SAA response only in young horses after endurance training. Arfuso et al. (2020) found an SAA increase in intensively trained jumpers, and Turlo et al. (2016) found an increase in racehorses after racing. Valle et al. (2015) found a twofold increase in eight eventing horses 10 min after the end of a two‐star cross‐country phase, but this can be easily explained by the very low pre‐ride values and easily put into perspective by the small sample size.

For WBC, there are at least three different causes that can lead to the observed slight increase after the end of exercise. First, muscle microdamage could lead to local inflammatory responses (Horohov et al., 2012; Mihelic et al., 2022). Second, increased sympathetic activity due to emotional stress could lead to leukocyte activation (Muñoz et al., 1999; Mihelic et al., 2022). Third, an increase in WBC may also arise from splenic contraction (Carakostas et al., 1981; Muñoz et al., 1999; McGowan, 2008).

As HCT and RBC increases in the same horses also indicate splenic contraction (Giers et al., 2023), at least part of the WBC increase is attributable to this. The theory that the WBC increase results from an activation of WBC from the vascular surface is supported by the increase being measured immediately after the end of exercise. An inflammatory response in the muscles is supported by the increase in muscle enzymes, which provides an indication of muscle microdamage (Giers et al., 2023) and by the distinct iron decrease the next morning, which is also seen as a sign of acute inflammation (Borges et al., 2007). Plasma shift adjustment shows that the WBC concentration in fact does not peak until 10 min after the end of exercise, so one could assume that not all of the increase is due to splenic contraction and WBC activation. But the fact that SAA, as a significantly more sensitive parameter, also for local inflammatory processes (Long & Nolen‐Walston, 2020), does not increase until the next morning speaks against an inflammatory reaction in the entirety of the horses. Based on the observed parameters (SAA, WBC and iron), indications of inflammation are subtle.

The authors believe that pre‐ride‐SAA could still be interesting to assess the inflammatory status of horses because training adaptations could potentially prevent injuries or falls (Witkowska‐Pilaszewicz et al., 2019). This approach has been explored for endurance horses (Cywinska et al., 2010), and above a cut‐off value of 1000 µg/mL before exercise, no horse could finish a long distance. We suggest further research on pre‐ride SAA levels and injury or fall rates in eventing horses to reduce the risk of injury to horses in eventing competitions.

### 4.7 Limitations

The variability in the time span between 30 min post‐exercise and the following morning (ranging from 11 to 21 h) is likely to have an impact on the results.

The selection of blood parameters in this study was constrained to well‐established ones, which means that a comprehensive examination of organ systems was not undertaken. There are many newer, but currently less established, blood parameters that are also informative for the analysed physiological processes. To assess oxidative stress, we measured an antioxidant enzyme, SOD, but the redox status of the horses was not determined.

One limitation of this study was the small number of horses, which was insufficient for establishing reference values in accordance with the American Society for Veterinary Clinical Pathology guidelines for determining reference intervals in animal species (Friedrichs et al., 2012).

Exercise intensity was not determined using additional, non‐blood‐based parameters such as heart rate, which would have improved the interpretation of changes in blood values.

Another significant limitation of the study is that the statistical analysis was limited to mean values, thus not allowing for the identification of individual horses deviating from these mean values. Nevertheless, this study provides a crucial foundation for personalized assessments.

## 5 CONCLUSION

In summary, increased levels of HGB and cortisol above the reference range occur regularly 10 and 30 min post‐exercises in trained eventing horses after difficult international competitions with submaximal exercise intensity. However, there are also physiological changes within the reference ranges. The HPA axis and the HPT axis are activated, and there is antioxidant activity. The increased energy demand during exercise leads to the mobilization of energy reserves, and a persistent increase in liver enzymes indicates hepatocellular injury. Signs of haemolysis reflect increased muscle metabolism during exercise. Indications of inflammation are subtle. Further research is needed to identify which horses deviate from mean values.

## AUTHOR CONTRIBUTIONS

*Conceptualization; methodology*: Katharina Kirsch, Stephanie Horstmann and Heidrun Gehlen. *Software*: Alexander Bartel. *Validation*: Alexander Bartel, Katharina Kirsch, Simon Franz Müller and Heidrun Gehlen. *Formal analysis; data curation; visualization*: Alexander Bartel and Johanna Giers. *Investigation; writing – original draft preparation*: Johanna Giers. *Resources*: Katharina Kirsch, Simon Franz Müller and Heidrun Gehlen. *Writing – review and editing*: Katharina Kirsch, Alexander Bartel, Simon Franz Müller, Stephanie Horstmann and Heidrun Gehlen. *Supervision; project administration*: Heidrun Gehlen. *Funding acquisition*: Katharina Kirsch and Heidrun Gehlen. All authors have read and agreed to the published version of the manuscript.

## CONFLICT OF INTEREST STATEMENT

S.F.M. is employed at the contributing commercial veterinary diagnostics laboratory, LABOKLIN GmbH & Co. KG as head of research and development of the department of clinical pathology, but has no role in the design of the study, in the collection of samples, in the interpretation of data or in the decision to publish the results. The authors declare no conflicts of interest.

## INSTITUTIONAL REVIEW BOARD STATEMENT

The study was registered with the regulatory state office of Berlin (1‐02.04.40.2022.VG006) but was not classified as an animal experiment.

## INFORMED CONSENT STATEMENT

Riders and owners received written information about the study conditions and agreed in writing to the voluntary and unremunerated participation of their horses.

## ANIMAL WELFARE STATEMENT

The study was registered with the regulatory state office of Berlin (1‐02.04.40.2022.VG006) but was not classified as an animal experiment. The authors confirm that the ethical policies of the journal, as noted on the journal's author guidelines page, have been adhered to.

### PEER REVIEW

The peer review history for this article is available at https://publons.com/publon/10.1002/vms3.1409.

## ETHICS STATEMENT

The authors confirm that the ethical policies of the journal, as noted on the journal's author guidelines page, have been adhered to and the appropriate ethical review committee approval has been received.

## Supporting information

Supporting Information

## Data Availability

The data presented in this study are available on request from the corresponding author. The data is not publicly available due to privacy.
