# Supplementary material for: Blood‐based assessment of oxidative stress, inflammation, endocrine and metabolic adaptations in eventing horses accounting for plasma volume shift after exercise
Source: Vet Med Sci. 2024 Mar 22;10(3):e1409. doi: 10.1002/vms3.1409 (PMC10958401; doi:10.1002/vms3.1409)
Supplement: Supplementary file 1 — Supporting Information [file VMS3-10-e1409-s001.docx]

Supporting Information

Title: Blood-based assessment of oxidative stress, inflammation, endocrine and metabolic adaptations in eventing horses accounting for plasma volume shift after exercise

Running title: Blood-based markers in eventing horses

**Table S1.** shows the reference ranges, units, and median (25th/ 75th quantiles) per time point.

| **Parameter** | **N** | **Missing** | **Reference range** | **Unit** | **Median (25th,75th)** | | | |
| --- | --- | --- | --- | --- | --- | --- | --- | --- |
|  |  |  |  |  | **Pre (n=55)** | **10 min (n=55)** | **30 min (n=55)** | **next morning (n=54)** |
| α-Amylase | 162 | 2 | < 50 | U/l | 4 (3;5) | n.m. | 5 (5;7) | 4 (3;5) |
| ALT | 162 | 2 | < 70 | U/l | 6.5 (5.3;7.8) | n.m. | 8.0 (6.4;9.7) | 7.7 (5.9;9.3) |
| AP | 162 | 2 | < 450 | U/l | 99 (72;133) | n.m. | 114 (82;144) | 109 (84;142) |
| Bilirubin | 162 | 2 | 8.6 - 59.9 | µmol/l | 25.3 (21.8;31.4) | n.m. | 29.5 (23.7;35.6) | 26.8 (23.8;35.1) |
| Cholesterol | 162 | 2 | 1.8 - 4.7 | mmol/l | 2.4 (2.3;2.6) | n.m. | 2.5 (2.4;2.7) | 2.5 (2.3;2.6) |
| Copper | 162 | 2 | 7.9 - 21.0 | µmol/l | 13.9 (11.8;15.9) | n.m. | 14.6 (12.6;17.4) | 14.0 (11.6;16.3) |
| Cortisol | 217 | 2 | < 50 | ng/ml | 42.4 (35.7;49.5) | 70.9 (65.5;77.8) | 64.2 (59.7;75.95) | 37.9 (29.1;44.4) |
| DGGR-Lipase | 162 | 2 | < 20 | U/l | 15.4 (13.5;17.3) | n.m. | 15.7 (13.9;18.2) | 15.9 (13.5;17.9) |
| GGT | 162 | 2 | < 25 | U/l | 11.3 (8.1;17.7) | n.m. | 13.3 (9.5;19.3) | 13.0 (9.4;17.9) |
| GLDH | 162 | 2 | < 8 | U/l | 1.3 (0.9;1.8) | n.m. | 2.2 (1.3;3.5) | 2.1 (1.6;2.7) |
| Globulines | 162 | 2 | < 51 | g/l | 27.2 (24.9;30.4) | n.m. | 28.5 (25.6;31.0) | 28.0 (24.7;30.0) |
| HGB | 219 | 0 | 107 - 165 | g/L | 129 (124;135) | 182 (174;191) | 160 (153;167) | 131 (124;139) |
| Iron | 162 | 2 | 17.9 - 64.5 | µmol/l | 22.9 (19.6;27.2) | n.m. | 27.5 (23.2;30.3) | 20.8 (17.7;24.0) |
| SAA | 162 | 2 | < 7.0 | µg/ml | 5.6 (4.0;6.7) | n.m. | 3.9 (2.3;6.6) | 3.5 (1.9;6.6) |
| Selenium | 162 | 2 | 100 - 200 | µg/l | 148.8 (126.3;169.2) | n.m. | 156.0 (139.7;185.8) | 157.5 (130.0;169.2) |
| SOD | 217 | 2 | n.a. | U/ml | 1.70 (1.09;2.37) | 2.79 (1.49;4.28) | 2.37 (1.48;3.07) | 2.07 (1.17;3.46) |
| T3 | 161 | 3 | 25 - 180 | ng/dl | 53.5 (44.9;69.1) | n.m. | 83.2 (62.6;99.3) | 61.0 (49.0;68.6) |
| T4 | 162 | 2 | 1.3 - 4.1 | µg/dl | 1.7 (1.4;2.0) | n.m. | 2.0 (1.7;2.6) | 1.7 (1.4;2.1) |
| Triglyceride | 162 | 2 | < 0.97 | mmol/l | 0.31 (0.27;0.35) | n.m. | 0.37 (0.28;0.46) | 0.31 (0.27;0.36) |
| Vitamin E | 162 | 2 | > 1.0 | mg/l | 3.5 (2.8;4.9) | n.m. | 4.0 (3.1;4.9) | 4.0 (3.0;5.1) |
| WBC | 219 | 0 | 4.90 - 11.10 | x10^9^/L | 7.04 (6.31;8.43) | 9.04 (8.02;9.99) | 8.46 (7.70;9.29) | 7.66 (6.91;8.91) |
| Zinc | 162 | 2 | 9.2 - 19.9 | µmol/l | 13.3 (11.1;14.6) | n.m. | 12.3 (10.0;14.5) | 12.2 (10.1;14.1) |

* Parameters in an alphabetical order; n.m. = not measured; n.a. = not available

**Table S2.** shows reference ranges, units, estimated marginal mean and 95 % confidence interval per time point for unadjusted values.

| **Parameter** | **Reference range** | **Unit** | **EMM + 95 % confidence interval** | | | |
| --- | --- | --- | --- | --- | --- | --- |
|  |  |  | **Pre** | **10 min** | **30 min** | **next morning** |
| α-Amylase | < 50 | U/l | 4 (3;5) | n.m. | 5 (5;6) | 4 (3;5) |
| ALT | < 70 | U/l | 6.9 (6.0;8.1) | n.m. | 8.4 (7.2;9.7) | 8.0 (6.9;9.2) |
| AP | < 450 | U/l | 120 (94;147) | n.m. | 129 (103;156) | 128 (102;155) |
| Bilirubin | 8.6 - 59.9 | µmol/l | 26.0 (22.2;29.7) | n.m. | 30.0 (26.3;33.8) | 29.1 (25.4;32.9) |
| Cholesterol | 1.8 - 4.7 | mmol/l | 2.5 (2.3;2.6) | n.m. | 2.6 (2.4;2.7) | 2.5 (2.3;2.7) |
| Copper | 7.9 - 21.0 | µmol/l | 14.5 (13.0;16.1) | n.m. | 15.2 (13.7;16.7) | 14.5 (13.0;16.1) |
| Cortisol | < 50 | ng/ml | 42.6 (38.3;46.9) | 71.5 (67.3;75.8) | 68.3 (64.0;72.6) | 37.3 (33.0;41.6) |
| DGGR-Lipase | < 20 | U/l | 15.9 (14.2;17.6) | n.m. | 16.1 (14.4;17.8) | 16.2 (14.5;17.9) |
| GGT | < 25 | U/l | 13.8 (11.0;17.4) | n.m. | 15.5 (12.0;19.5) | 15.1 (12.0;19.1) |
| GLDH | < 8 | U/l | 1.3 (1.0;1.8) | n.m. | 2.3 (1.7;3.2) | 2.3 (1.7;3.1) |
| Globulines | < 51 | g/l | 28.0 (26.2;29.8) | n.m. | 29.0 (27.2;30.7) | 28.0 (26.2;29.8) |
| HGB | 107 - 165 | g/L | 129 (124;134) | 183 (178;188) | 161 (156;166) | 131 (126;136) |
| Iron | 17.9 - 64.5 | µmol/l | 23.9 (21.8;25.9) | n.m. | 27.3 (25.3;29.3) | 21.3 (19.3;23.4) |
| SAA | < 7.0 | µg/ml | 5.65 (3.21;9.50) | n.m. | 4.62 (2.57;7.83) | 4.27 (2.35;7.30) |
| Selenium | 100 - 200 | µg/l | 149 (136;162) | n.m. | 159 (146;172) | 150 (137;163) |
| SOD | n.a. | U/ml | 1.56 (1.18;2.07) | 2.22 (1.68;2.94) | 2.15 (1.63;2.84) | 1.87 (1.42;2.48) |
| T3 | 25 - 180 | ng/dl | 55.0 (49.0;63.1) | n.m. | 79.4 (70.8;89.1) | 56.2 (50.1;64.6) |
| T4 | 1.3 - 4.1 | µg/dl | 1.6 (1.4;1.9) | n.m. | 2.0 (1.8;2.2) | 1.6 (1.4;1.8) |
| Triglyceride | < 0.97 | mmol/l | 0.31 (0.27;0.35) | n.m. | 0.39 (0.35;0.44) | 0.32 (0.28;0.36) |
| Vitamin E | > 1.0 | mg/l | 3.6 (3.0;4.2) | n.m. | 3.8 (3.2;4.4) | 3.9 (3.2;4.5) |
| WBC | 4.90 - 11.10 | x10^9^/L | 7.49 (6.69;8.28) | 9.40 (8.60;10.19) | 8.92 (8.12;9.71) | 8.17 (7.37;8.96) |
| Zinc | 9.2 - 19.9 | µmol/l | 13.1 (11.9;14.3) | n.m. | 12.7 (11.5;14.0) | 12.5 (11.3;13.7) |

* Parameters in an alphabetical order; n.m. = not measured; n.a. = not available
